# Characterizing Alcohol Use and Expectancies for Alcohol Analgesia Among Breast Cancer Survivors With Arthralgia

**DOI:** 10.1002/pon.70517

**Published:** 2026-06-18

**Authors:** Jessica M. Powers, Lisa R. LaRowe, Tamara J. Somers, Judith A. Paice, Francis J. Keefe, Rebecca A. Shelby, Betina Yanez, Gretchen G. Kimmick, Christine Rini

**Affiliations:** ^1^ Department of Psychology University of Kansas Lawrence Kansas USA; ^2^ Cofrin Logan Center for Addiction Research & Treatment University of Kansas Lawrence Kansas USA; ^3^ University of Kansas Cancer Center Kansas City Kansas USA; ^4^ Mongan Institute Center for Optimal Aging and Serious Illness Massachusetts General Hospital Boston Massachusetts USA; ^5^ Department of Medicine Harvard Medical School Boston Massachusetts USA; ^6^ Department of Psychiatry and Behavioral Sciences Duke University School of Medicine Durham North Carolina USA; ^7^ Division of Hematology‐Oncology Northwestern University Feinberg School of Medicine Chicago Illinois USA; ^8^ Robert H. Lurie Comprehensive Cancer Center of Northwestern University Chicago Illinois USA; ^9^ Pain Prevention and Treatment Research Program Duke University Durham North Carolina USA; ^10^ Department of Medical Social Sciences Northwestern University Feinberg School of Medicine Chicago Illinois USA; ^11^ Duke Cancer Institute Durham North Carolina USA

**Keywords:** alcohol drinking, cancer survivors, pain

## Abstract

**Background:**

Expectancies for alcohol analgesia (EAA; beliefs that alcohol use can reduce pain) are linked to heavier drinking in chronic non‐cancer pain; however, no studies have examined EAA in those with cancer‐related pain.

**Aims:**

These secondary analyses examined cross‐sectional associations among EAA, alcohol use, and pain among breast cancer survivors with persistent pain due to aromatase inhibitors (AIs; arthralgia).

**Methods:**

Data were from 213 female breast cancer survivors with AI‐associated arthralgia (M age = 58.9, 77.5% White) who completed baseline assessment in a behavioral pain intervention trial. Alcohol consumption and alcohol use severity were assessed via the Alcohol Use Disorders Identification Test (AUDIT). Linear/logistic regressions were conducted covarying for depression, anxiety, age, race, and pain severity.

**Results:**

EAA scores were related to greater likelihood of current drinking (*AOR* = 6.02, *p* = 0.003). Among participants who reported current alcohol use (*n* = 153), EAA scores were associated with greater alcohol consumption and alcohol use severity (*p*s < 0.001). Probing of a significant pain severity x EAA interaction (*p* = 0.015) indicated that EAA scores were more strongly associated with alcohol use severity among participants with less (vs. more) severe pain.

**Conclusions:**

In breast cancer survivors with AI‐associated arthralgia, EAA are related to greater likelihood of drinking and heavier/more problematic alcohol use, which may reduce efficacy of AIs while increasing risk for side effects. EAA scores were more strongly associated with drinking in women with less severe pain.

## 1 Introduction

Expectancies for alcohol analgesia are beliefs or expectations that alcohol use can reduce or relieve pain. A growing body of evidence demonstrates that—regardless of the presence of a chronic pain condition—individuals who hold greater expectancies for alcohol analgesia are more likely to engage in heavier/hazardous alcohol use, drink in response to pain, and hold other maladaptive beliefs regarding alcohol use [1, 2]. Indeed, decades of research show that alcohol use is impacted by individuals' beliefs about outcomes of drinking [3], including beliefs about how drinking affects pain [1]. A growing number of laboratory studies have shown that greater expectancies for alcohol analgesia predict stronger perceived pain relief from alcohol during acute pain induction [4, 5], Likewise, numerous cross‐sectional studies have linked greater expectancies for alcohol analgesia to higher frequency of drinking, greater overall alcohol consumption, urge to drink, other alcohol outcome expectancies (e.g., positive and negative effects of alcohol), and greater reported pain severity [6, 7, 8, 9, 10, 11].

Theoretical frameworks suggest that alcohol use and pain interact in a bidirectional manner [12]. Specifically, these frameworks highlight that pain is a powerful motivator to drink due to short‐term analgesic effects of alcohol; however, alcohol use can worsen pain over time [13]. Expectancies for alcohol analgesia have been identified as an important mechanism that may underlie the relationship between pain and alcohol use [2, 14]. For example, the CANUE model (Catastrophizing, Anxiety, Negative Urgency, and Expectancy) posits that individuals with stronger expectancies for substance‐related analgesia are more likely to engage in substance use as a way to cope with pain and pain‐related negative affect [1].

All research on expectancies for alcohol analgesia has been conducted in either general population samples or in chronic non‐cancer pain. However, pain is a common symptom of numerous chronic medical conditions, including cancer [15]. For instance, over 40% of breast cancer survivors report persistent pain [16]. Postmenopausal breast cancer survivors with hormone receptor positive tumors are commonly prescribed aromatase inhibitors (AIs) as a standard adjuvant endocrine therapy to significantly reduce cancer recurrence and improve survival rates [17]. A significant and most commonly reported negative side effect of AIs is arthralgia [17], broadly defined as joint and bone pain and stiffness, in addition to myalgia (muscle pain). About 70% of women experience moderate to severe symptoms [18], which can interfere with daily functioning, increase distress, and reduce health‐related quality of life [19].

Cancer‐related pain is often considered separately from chronic non‐cancer pain because it arises from distinct etiologies (e.g., tumor involvement, treatment side effects, disease progression), follows a different clinical trajectory, and may require specialized treatment approaches embedded within the broader context of cancer survivorship care. Despite pain being a common symptom in breast cancer survivors, limited work has examined pain and alcohol use in this population, and we are unaware of any prior research in this area conducted in AI‐associated arthralgia. Alcohol use is discouraged in the context of breast cancer due to impact on treatment side effects and increasing risk for cancer recurrence [20]. Drinking is also generally not recommended on AIs [21], with ongoing studies examining whether alcohol use impacts changes in sex hormone levels potentially impairing drug metabolism [22]. It is therefore important to characterize alcohol use in this population. Although findings by members of our research team suggest that cancer survivors with pain (vs. no pain) may be less likely to endorse alcohol use [23], evidence has also shown that cancer‐related pain is a feared symptom, in part due to concerns that it may signal a return or advance of cancer [24]. Fears of cancer recurrence have been linked to heavier and increased alcohol consumption among cancer survivors post‐diagnosis, potentially in an effort to cope with related emotional distress [25]. Thus, there is a further need to examine pain and alcohol use in cancer survivorship, including among breast cancer survivors with AI‐associated arthralgia, in addition to identifying factors such as expectancies for alcohol analgesia that may contribute to heavier alcohol use and more maladaptive pain coping.

Thus, the goals of the current secondary analysis were (1) to characterize alcohol use among breast cancer survivors with AI‐associated arthralgia, (2) to examine associations between pain severity, expectancies for alcohol analgesia, and measures of alcohol use severity and consumption, and (3) to explore the interaction between pain severity and expectancies for alcohol analgesia on alcohol use. Data were drawn from the baseline (pre‐randomization) assessment of a randomized controlled trial (RCT) testing the efficacy of an online pain coping skills training intervention (NCT05703178). We hypothesized that greater expectancies for alcohol analgesia would be associated with greater alcohol use severity and alcohol consumption in the sample. We also examined whether pain severity moderated this relationship and hypothesized that expectancies for alcohol analgesia would be more strongly associated with alcohol intake among breast cancer survivors who experienced more severe pain.

## 2 Method

### 2.1 Participants

Data was drawn from the baseline assessment of the Web‐based pain coping SKills training to Improve Pain (SKIP)‐Arthralgia trial, which is examining the efficacy of an online pain coping skills training program for managing AI‐associated arthralgia in female breast cancer survivors. Participants were English‐speaking women aged ≥ 18 years who were diagnosed with Stage I–III, hormone receptor‐positive (HR+) breast cancer, had completed primary cancer treatment and were currently using AI therapy. Pain criteria included at least 15 days of pain in the past 30 days and a worst pain rating of ≥ 4 on an 11 point (0–10) numerical rating scale in the past week. Participants could have a pre‐existing chronic pain condition (e.g., osteoarthritis); however, eligibility criteria included pain that developed or worsened since starting AI therapy. Participants were recruited from two study sites (Northwestern University, Duke University) and the trial was registered with ClinicalTrials.gov (NCT05703178). The study was approved by the IRB at Northwestern University (lead site). Informed consent was obtained from all individual participants in the study. Additional study details can be found elsewhere [26]. The current secondary analyses included the 213 participants who completed measures of EAA and alcohol use. A subset of analyses was also conducted among the 153 participants who endorsed current alcohol use over the past year.

### 2.2 Measures

#### 2.2.1 Expectancies for Alcohol Analgesia

Expectations for alcohol‐related pain inhibition were assessed using the five‐item measure of Expectancies for Alcohol Analgesia (EAA) [6]. Items assess the perceived likelihood that drinking alcohol will reduce or help cope with pain, with responses ranging from 0 (Completely Unlikely) to 9 (Completely Likely). Prior work has demonstrated the validity and reliability of the EAA among adults with chronic pain [6]. Responses are summed to generate a total score (Range: 0–45). The EAA had excellent internal consistency (*α* = 0.96).

#### 2.2.2 Current Alcohol Use, Alcohol Use Severity, and Alcohol Consumption

The 10‐item Alcohol Use Disorders Identification Test (AUDIT) was used to assess current alcohol use, alcohol consumption, and alcohol use severity [27]. The first item assesses current drinking (“How often do you have a drink containing alcohol?”). Current alcohol use was defined as participants who endorsed drinking at least monthly or less versus never in the past year. The first three items of the AUDIT assess frequency/quantity of alcohol consumption and are used to calculate the alcohol consumption subscale (AUDIT‐C; Range: 0–12). The remaining seven items assess frequency of alcohol use problems over the last year (e.g., “How often during the last year have you failed to do what was normally expected from you because of drinking?”). All 10 AUDIT items are summed to generate a total score of overall alcohol use severity (Range: 0–40), with higher scores indicating greater severity. For the current analyses, alcohol use severity and consumption were calculated among participants who reported current alcohol use (*n* = 153).

#### 2.2.3 Pain Severity

The Brief Pain Inventory (BPI) short form was used to assess pain severity over the past week, using the pain severity subscale [28]. Pain severity includes four items assessing worst, least, average, and current pain severity. Responses are provided on an 11‐point scale ranging from 0 (“No pain”) to 10 (“Pain as bad as you can imagine”). Responses are averaged (Range: 0–10), with higher scores indicating greater pain severity.

#### 2.2.4 Anxiety and Depression Symptoms

The Hospital Depression and Anxiety Scale (HADS) was used to assess anxiety (7 items) and depression symptoms (7‐items) in the sample [29]. The HADS generates separate subscales for anxiety and depression. Responses for each subscale are summed to generate depressive symptoms and anxiety scores ranging from 0 to 21, with higher scores indicating greater frequency of depression or anxiety symptoms, respectively.

#### 2.2.5 Sociodemographic, Substance Use, and Participant Characteristics

Sociodemographic characteristics were self‐reported, including race, ethnicity, past‐year annual income, and age. Participants were also asked about cigarette smoking and electronic cigarette (e‐cigarette) use, and whether they used cannabis for sleep/pain relief in the past 7‐days (yes/no).

### 2.3 Data Analytic Plan

All analyses were conducted using SPSS Version 30.0. Data was first examined for normality, and EAA scores were log‐transformed due to skewness and kurtosis values exceeding recommended thresholds. After transformation, skewness and kurtosis values were appropriate (skew = 1.58, kurtosis = 1.03). We estimated associations between EAA and AUDIT scores, depressive symptoms, anxiety, pain medication use, and sociodemographic/participant characteristics using Pearson correlation for pairs of continuous variables and point biserial correlation for pairs including a continuous and dichotomous variable. Variables that were significantly correlated with EAA or AUDIT total or consumption scores were included as covariates in subsequent statistical models. Additional covariates were identified (i.e., anxiety, age) due to established associations with alcohol use and pain as demonstrated in the empirical literature. We then conducted logistic regression and hierarchical linear regression models to examine effects of covariates (Step 1), EAA scores and pain severity (Step 2), and the interaction between EAA scores x pain severity (Step 3) on alcohol outcomes (Current Alcohol Use, AUDIT Total Scores, AUDIT Consumption Scores). Models examining AUDIT Total Scores and Consumption scores were limited to *n* = 153 participants who endorsed current alcohol use. Significant interactions were probed by testing the conditional effects of EAA scores at three levels of pain severity/interference (−1 SD, *M*, + 1 SD). The Holman‐Bonferroni method was used to correct for multiple comparisons.

## 3 Results

### 3.1 Participant Characteristics

Among the total sample of *N* = 213, mean age was 58.99 years (SD = 10.28), and a majority of participants were White (77.5%) and married (69.5%). There was a high level of education, with 34.3% reporting a graduate‐level degree. Almost 30% of the sample reported a total household income of over $140,000 in the past year. More participants were recruited from Duke University (62.4%) than Northwestern University (37.6%). Only *n* = 5 participants reported using cannabis for pain/sleep in the past week. Additionally, only *n* = 2 participants reported current cigarette smoking and *n* = 3 current e‐cigarette use. All participant characteristics are presented in Table 1.

**TABLE 1:** Sample characteristics.

	Total sample (*N* = 213)
	% (*N*)
Race	
White	77.5% (165)
Black	12.2% (26)
Other	8.0% (2)
Hispanic/Latino ethnicity	2.3% (5)
Education level	
Some high school	0.9% (2)
High school/GED	3.3% (7)
Some college/Technical school	15.4% (33)
2 or 4‐year college degree	45.5% (97)
Graduate degree	34.3% (73)
Employment status	
Full‐time	46.0% (98)
Part‐time	10.3% (22)
Retired or unemployed	43.7% (93)
Past‐year income	
< $40,000	9.0% (19)
$40,000–$79,999	14.5% (31)
$80,000–$119,999	18.5% (39)
$120,000–$139,999	8.5% (18)
> $140,000	29.5% (63)
Prefer not to answer	19.2% (41)
Study site	
Northwestern university	37.6% (80)
Duke university	62.4% (133)
Cancer stage	
Stage I–II	90.6% (193)
Stage III	9.4% (20)

	*M* (SD)
Age (years)	58.99 (10.28)
BPI—Pain severity	4.16 (1.50)
HADS—Anxiety	6.50 (4.12)
HADS—Depression	4.91 (3.39)
EAA	3.20 (7.60)
AUDIT—Alcohol use severity	2.01 (2.43)
AUDIT—Consumption	1.71 (1.75)

*Note:* Percentages may not add up to 100% due to missing data or non‐response.

### 3.2 Characterizing Alcohol Use and Expectancies for Alcohol Analgesia

Approximately 29% of the sample endorsed never drinking alcohol (*n* = 61). Among those who reported current drinking, 29.1% reported drinking monthly or less, followed by 2–3 times a week (17.8%), 2–4 times per month (16.4%) and 4 or more times a week (8.0%). Only *n* = 10 met criteria for hazardous drinking (AUDIT total score ≥ 8). Among the 153 participants who reported alcohol use, almost all (90.2%) reported typically consuming 1–2 drinks per occasion. Among the total sample, AUDIT consumption (*M* = 1.71, SD = 1.75; Range: 0–9) and Alcohol Use Severity (*M* = 2.01, SD = 2.43; Range: 0–16) were low (see Table 1).

Overall, the average EAA score was 3.20 (SD = 7.60), with almost 75% of participants (*N* = 157) endorsing scores of 0 (Range: 0–45). Among participants who endorsed never drinking (*n* = 71), the average EAA score was 1.01 (SD = 5.46). Among participants who endorsed current drinking (*n* = 153), the average EAA score was 4.08 (SD = 8.16). In the total sample, EAA scores were not associated with sociodemographic characteristics, pain medication use, anxiety medication use, anxiety, depressive symptoms, or cancer stage. Alcohol consumption scores were significantly associated with lower depression scores (*r* = −0.16, *p* = 0.019) and race (*p* = 0.044). Probing using a one‐way ANOVA revealed that White participants endorsed greater alcohol consumption (*M* = 1.92, SD = 1.81; *p* = 0.002) than Black participants (*M* = 0.81, SD = 1.06). Thus, depression and race were included as covariates in subsequent adjusted models, in addition to age and anxiety.

### 3.3 Examining Expectancies for Alcohol Analgesia and Pain Severity in Relation to Current Alcohol Use and Alcohol Use Severity and Consumption

#### 3.3.1 Current Alcohol Use

Pain severity was associated with lower likelihood of current alcohol use (*AOR* = 0.75, *p* = 0.020; see Table 2), whereas expectancies for alcohol analgesia were associated with greater likelihood (*AOR* = 6.02, *p* = 0.003), Specifically, every 1‐point increase in EAA scores was associated with 6.02 times higher odds of endorsing current alcohol use. There was no significant EAA x pain severity interaction on likelihood of current alcohol use (*p* = 0.589).

**TABLE 2:** Logistic regression model examining pain severity and expectancies for alcohol analgesia in relation to current alcohol use.

	Current alcohol use		
	AOR	[95% CI]	*p*
Step 1			
Age	0.98	0.95–1.01	0.987
Race			
White [REF]	—	—	—
Black	0.26	0.11–0.63	0.003*
Other	0.43	0.15–1.25	0.123
Anxiety^a^	0.97	0.95–1.01	0.186
Depression^b^	0.95	0.85–1.06	0.949
Step 2			
Pain severity^c^	0.75	0.60–0.96	0.020*
EAA^d^	6.02	1.88–19.31	0.003*
Step 3			
Pain severity x EAA	0.60	0.23–1.49	0.265

^a^ Hospital Anxiety and Depression Scale (HADS)—Anxiety Subscale.

^b^ Hospital Anxiety and Depression Scale (HADS)—Depression Subscale.

^c^ Brief Pain Inventory (BPI)—Pain Severity Subscale.

^d^ Expectancies for Alcohol Analgesia (EAA).

^*^ *p* < 0.05.

#### 3.3.2 Alcohol Use Severity and Consumption

**FIGURE 1:**
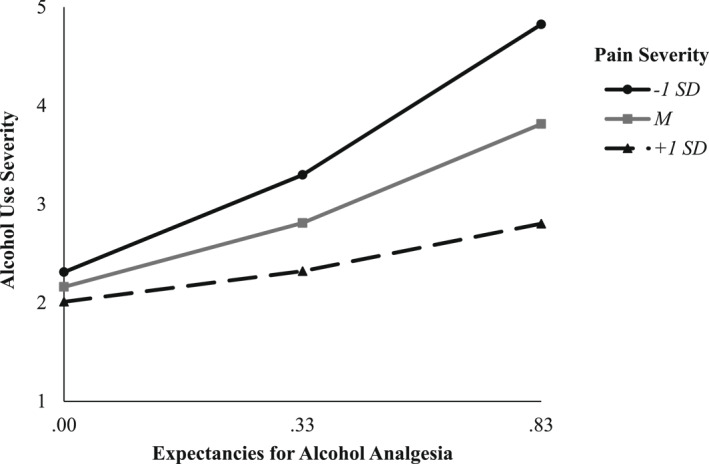
Conditional effects of pain severity on associations between expectancies for alcohol analgesia (eaa) and alcohol use severity. Pain severity subscale of the brief pain inventory (BPI); Levels of pain severity probed include: −1 SD, M, +1 SD; Alcohol use severity assessed via total scores on the alcohol use disorders identification test (AUDIT); EAA scores were log transformed; Limited to participants who reported current alcohol use (*n* = 153).

Models examining alcohol use severity and alcohol consumption were conducted among *N* = 153 who endorsed current alcohol use (Table 3). Results demonstrated main effects of EAA scores (Step 2: *β* = 0.41, *p* < 0.001) on alcohol use severity, such that greater expectancies for alcohol analgesia were associated with greater alcohol use severity, after controlling for effects of covariates (Step 1) and pain severity (Step 2). There was a borderline, but non‐significant main effect of pain severity on alcohol use severity (*p* = 0.056). In Step 3, a significant EAA x pain severity interaction was observed (*p* = 0.015). Probing of these conditional effects revealed that expectancies for alcohol analgesia were more strongly associated with alcohol use severity among participants with lower levels of pain severity (−1 SD: 2.51; *b* = 3.01, SE = 0.56, *p* < 0.001) whereas there was no significant association at higher levels of pain severity (+1 SD: 5.32; *b* = 0.95, SE = 0.53, *p* = 0.077; see Figure 1).

**TABLE 3:** Hierarchical linear regression models examining expectancies for alcohol analgesia, pain severity/interference, and alcohol use severity and consumption among participants who endorsed current alcohol use (*n* = 153).

	Alcohol use severity					Alcohol consumption				
	*β*	*t*	*p*	Δ*R* ^2^	*p* for Δ*R* ^2^	*β*	*t*	*p*	Δ*R* ^2^	*p* for Δ*R* ^2^
Step 1				0.06	0.096				0.08	0.046*
Age	0.05	0.53	0.596			0.08	0.880	0.380		
Race										
White [REF]										
Black	−0.12	−1.52	0.132			−0.12	−1.51	0.134		
Other	−0.04	−0.47	0.639			−0.11	−1.26	0.209		
Anxiety^a^	0.24	2.40	0.017*			0.22	2.22	0.028*		
Depression^b^	−0.20	−2.14	0.034*			−0.24	−2.57	0.011*		
Step 2				0.17	< 0.001**				0.14	< 0.001**
Pain severity^c^	−0.15	−1.92	0.057			−0.16	−2.03	0.044*		
EAA^d^	0.41	5.36	< 0.001**			0.36	4.65	< 0.001**		
Step 3				0.03	0.015*				0.01	0.129
Pain severity x EAA	−0.62	−2.45	0.015*			−0.40	−1.01	0.129		

^a^ Hospital Anxiety and Depression Scale (HADS)—Anxiety Subscale.

^b^ Hospital Anxiety and Depression Scale (HADS)—Depression Subscale.

^c^ Brief Pain Inventory (BPI)—Pain Severity Subscale.

^d^ Expectancies for Alcohol Analgesia (EAA); Alcohol Use Severity and Alcohol Consumption assessed via Alcohol Use Disorders Identification Test (AUDIT).

^*^ *p* < 0.05.

^**^ *p* < 0.001.

Models examining alcohol consumption showed main effects of both expectancies for alcohol analgesia (Step 2: *β* = 0.36, *p* < 0.001) and pain severity (Step 2: *β* = −0.16, *p* = 0.044; Table 3). Specifically, greater expectancies for alcohol analgesia and greater pain severity were each associated with greater reported alcohol consumption among individuals who endorsed current alcohol use. There was no significant EAA x pain severity (*p* = 0.129) interaction at Step 3 of the model.

## 4 Discussion

This is the first study to examine expectancies for alcohol analgesia and alcohol use among breast cancer survivors with AI‐associated arthralgia. Findings indicate that women in the sample endorsed relatively low levels of alcohol use (29% reported never drinking in the past year) and low levels of expectancies for alcohol analgesia. However, results also demonstrated that greater expectancies for alcohol analgesia were associated with greater likelihood of reporting current alcohol use, in addition to greater alcohol use severity and heavier overall alcohol consumption among participants who endorsed current drinking. There was a significant interaction in models examining alcohol use severity. Probing of these effects revealed that expectancies for alcohol analgesia were more strongly related to alcohol use severity among women with lower pain severity versus those with moderate or high pain severity. However, effects of this interaction should be interpreted with caution due to the large percentage (75% of the overall sample) of participants who endorsed scores of 0 on the EAA.

These findings build upon prior literature showing that holding greater expectations that alcohol use will provide pain relief is related to heavier and more problematic alcohol use and greater likelihood of drinking in response to pain [4, 6, 10], while extending them to a new, clinically important population. It is notable that effects of expectancies in models were above and beyond variance accounted for by pain severity. This suggests that individuals' beliefs about the analgesic effects of alcohol are associated with alcohol use, independent of pain severity. Indeed, prior work demonstrates that expectancies for alcohol analgesia are related to quantity and frequency of alcohol consumption and the urge to drink among individuals without chronic pain [7]. The fact that findings were among breast cancer survivors with AI‐associated arthralgias have additional implications, given that alcohol use can likely worsen pain via dysregulation of the endogenous opioid system and that alcohol use is not recommended in the context of breast cancer survivorship [13, 20, 21].

Mean scores on the measure of expectancies for alcohol analgesia were quite low, and almost 75% of participants endorsed scores of 0, indicating that these participants felt it was completely unlikely that drinking alcohol would help with their pain. Our sample's mean expectancies scores were substantially lower than scores of individuals without chronic pain (*M* = 15.49) [7], and adults with chronic pain who endorsed regular alcohol use (*M* = 25.75) [6]. Of note, this is the first study of EAA that included individuals who do not drink alcohol, and the prevalence of non‐drinking in the sample was approximately 29%, which is lower than that of the general population [30]. We found that EAA scores were higher among participants who endorsed current alcohol use (vs. never drinking); however, it is still important to assess EAA in non‐alcohol use as individuals can develop alcohol expectancies due to social and/or cultural transmission. It is possible that the current results reflect differences in the prevalence of alcohol use and expectancies for alcohol analgesia among this population.

### 4.1 Implications

Consistent with this proposition, emerging evidence has found that cancer survivors with pain (vs. no pain) are less likely to drink alcohol [23]. There has also been increasing focus on negative effects of alcohol in breast cancer [20]. One clinical implication of these findings is that breast cancer survivors may be aware of these negative effects and thus reduce their alcohol consumption or hold more negative beliefs about alcohol use. Indeed, over half of breast cancer survivors reduce or stop drinking after receiving a diagnosis, with evidence showing a four to sixfold greater likelihood of alcohol cessation among survivors who received information/counseling from a medical provider [31]. One possibility is that cancer survivors with arthralgia may limit their alcohol consumption due to concerns about alcohol on risk of developing a secondary cancer or cancer recurrence, or exacerbation of vasomotor symptoms common in AI. Another possibility is that the current sample is well‐connected with treatment and may have received guidance from their medical provider regarding alcohol use in the context of cancer care, although we were unable to assess this in the current study. Alternatively, it is possible that participants underestimated and/or underreported their alcohol use and beliefs about the pain‐relieving effects of alcohol due to concerns about stigma and reluctance for their medical providers to know about their drinking. Further research is needed to elucidate patterns of alcohol use and pain among breast cancer survivors, including directly assessing positive and negative alcohol‐related attitudes/expectancies among cancer survivors. Clinicians should also incorporate routine assessment of alcohol use in the context of cancer treatment.

Additional implications include that expectancies for alcohol analgesia were related to greater alcohol use severity among participants with less severe pain, compared to those with more severe pain. These results should be interpreted with caution due to the small sample size. Though we initially expected that participants with greater pain severity and more expectancies for alcohol analgesia would report the most problematic alcohol use behavior based in previous theory [1], it makes some sense that expectancies for alcohol analgesia were most strongly related to drinking among those with lower (vs. higher) pain severity. Previous work has shown that pain is a strong motivator of drinking behavior [32], and that it is associated with more hazardous patterns of alcohol use [33, 34, 35]. Thus, individuals with more severe pain may be more likely to use alcohol regardless of their beliefs about its analgesic effects. In contrast, women experiencing lower levels of pain may have access to a broader repertoire of effective pain coping strategies and may only turn to alcohol if they have strong beliefs about its pain‐relieving effects. Additionally, women experiencing lower levels of pain may be less likely to perceive their pain as indicative of a serious problem, which could further increase their likelihood of turning to alcohol for self‐management rather than seeking formal care or other treatment alternatives.

Future work is needed to further explore this relationship in larger and more diverse samples of breast cancer survivors. Additional implications include ensuring breast cancer survivors have access to evidence‐based pain management strategies, to reduce alcohol use in the context of pain.

### 4.2 Study Limitations

There are several limitations that are important to acknowledge. First, the sample was primarily White, highly educated with high income, which have been shown to be related to lower levels of alcohol use and lower positive alcohol expectancies [36, 37]. Additional work has shown Future work is needed with more diverse samples in terms of sociodemographic and drinking characteristics. Second, the cross‐sectional nature of the current analyses limits causal determination. It is possible that expectancies for alcohol analgesia can lead to heavier drinking to cope with pain. Conversely, it is also possible that regular alcohol use can provide more opportunities to develop expectancies for alcohol analgesia. Evidence also suggests that heavier alcohol use over time is associated with greater pain [12]. Further research should incorporate prospective designs to evaluate the plausibility of causal relationships. In analyses not reported, we did not find any differences in outcomes as a function of pain medication use; however, this study was not designed to examine this possibility rigorously. For example, only *n* = 13 participants reported current use of opioid pain medications, and further research is needed to examine effects of pain/opioid medications. Finally, as mentioned above, it is possible that participants might underreport alcohol use or EAA given the context of completing a study in a medical center and given concerns about effects of drinking on pain treatment access. Further work incorporating methods such as qualitative data to assess participants beliefs and perceived stigma around alcohol use in the context of cancer and cancer‐related pain treatment could be beneficial to further elucidate these relationships.

## 5 Conclusion

This research demonstrates that breast cancer survivors with AI‐associated arthralgia generally report low levels of alcohol use and report low levels of expectations that drinking can help relieve pain. Furthermore, there were substantial variations in expectancies and those reporting greater expectancies for alcohol analgesia were much more likely to report drinking and heavier/more problematic alcohol use, and that these associations were stronger among survivors with less (vs. more) severe pain. Alcohol use can have a negative impact on pain [12]. Very preliminary work has also begun to examine whether alcohol may worsen side effects of AI medications [22], and alcohol consumption may increase risk of breast cancer recurrence [20]. Taken together, these findings highlight the need to assess both alcohol use and expectancies for alcohol analgesia among breast cancer patients with AI‐associated arthralgia to promote early identification of individuals at greater risk for poorer pain and health outcomes.

## Author Contributions

**Jessica M. Powers:** conceptualization, formal analysis, methodology, visualization, writing – original draft, writing – review and editing. **Lisa R. LaRowe:** methodology, writing – original draft, writing – review and editing. **Tamara J. Somers:** investigation, methodology, project administration, funding acquisition, writing – review and editing. **Judith A. Paice:** investigation, methodology, writing – review and editing. **Francis J. Keefe:** funding acquisition, investigation, methodology, writing – review and editing. **Rebecca A. Shelby:** investigation, methodology, project administration, writing – review and editing. **Betina Yanez:** investigation, writing – review and editing. **Gretchen G. Kimmick:** investigation, methodology, project administration, writing – review and editing. **Christine Rini:** funding acquisition, investigation, methodology, project administration, supervision, writing – original draft, writing – review and editing.

## Funding

J.M.P. was supported by the National Cancer Institute (T32CA193193). L.R.L. was supported by a K23 awarded by the National Institute on Aging (K23AG088376). Funding for the study that provided data used for this project was from the National Cancer Institute (R01CA271220, PI: Rini).

## Conflicts of Interest

GK reports with several years has had research funding from Pfizer, participated in clinical trials funded by Pharma to the Breast Medical Oncology Division, participated in Advisory Board for Boehringer Ingelheim, Eisai, Genomic Health and Agendia, and received royalties from UpToDate and Springer. Authors have no additional disclosures to report.
